# Evaluation of breast calcifications

**DOI:** 10.4103/0971-3026.57208

**Published:** 2009-11

**Authors:** Yojana V Nalawade

**Affiliations:** Asian institute of Oncology and S. L. Raheja Hospital, Nutan mammography Centre, 2- A Manubharti, Azad lane. S. V. Road, Andheri West, Mumbai, India

**Keywords:** Benign calcifications, microcalcifications, wire localization, malignant calcifications

## Abstract

Various patterns of calcifications occur in the breast; some benign, some malignant. A knowledge of these patterns on mammography helps in accurate interpretation and management.

## Introduction

Microcalcifications can be the early and only presenting sign of breast cancer. Mammography is used worldwide to detect microcalcifications. Hence, with the help of mammography, we can not only diagnose cancer in a nonpalpable stage but can also detect the extent of the disease. It is very essential to perform a proper evaluation of various calcifications to decide whether they are benign or malignant. A biopsy can be avoided if the calcifications appear absolutely benign on mammography and the patient can be followed-up with annual screening mammography.

In 1913, a German surgeon, Solomon, reported the presence of microcalcifications in the radiographic examination of a mastectomy specimen. In 1949, Leborgne, a radiologist, postulated that the presence of microcalcifications may be the only mammographic manifestation of a carcinoma.[1] Since then, all radiologists have made active efforts to look for microcalcifications in mammograms and this in turn over the years has resulted in a significant improvement in the resolution and performance of the mammography machines.

To detect microcalcifications efficiently, a good mammography machine should have:

- dedicated mammography grids,
- a small focal spot and
- a proper source image distance

In addition, the following are necessary:

- Magnification. Every area of microcalcifications should be magnified.
- Proper processing of the mammography films should be performed, with longer processing times as compared to conventional radiography.
- The use of a magnifying glass, which helps in better visualization, is a must.
- A dedicated mammography viewing box (more than 3000 nit) should be used.
- There should be very little (<50 lux) ambient light in the room.
- A computed-aided diagnosis (CAD) system is useful when evaluating a large volume of examinations, although CAD systems may sometimes fail to pick up amorphous calcifications.[2]

Full-field digital mammography machines are better than film-screen mammography machines for diagnosing microcalcifications. High-resolution computer radiography (CR) machines cannot detect microcalcifications efficiently.[3]

Once calcifications are detected, they have to be described and categorized according to the lexicon mentioned in BI-RADS (Breast Imaging Reporting And Data System) so that the radiologist, the surgeon and the pathologist share a common language. BI-RADS, developed by the American college of Radiology, is followed worldwide to describe and categorize breast abnormalities.

In the chapter titled ‘Lexicons’ in the official BI-RADS publication, calcifications are described according to their appearance and distribution.

### According to appearance

#### Calcifications that are typically benign are described as follows:[5]

##### Eggshell or rim-like calcifications:

**Figure 1:**
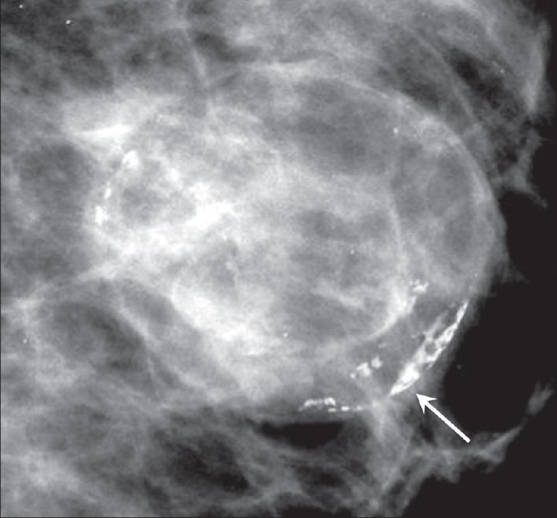
Mammogram shows rim/egg-shell calcification (arrow)

These are thin, round, rim-like calcifications often seen in the walls of cysts or in fat necrosis [Figure 1].

##### Coarse and popcorn-like calcifications:

**Figure 2 (A,B):**
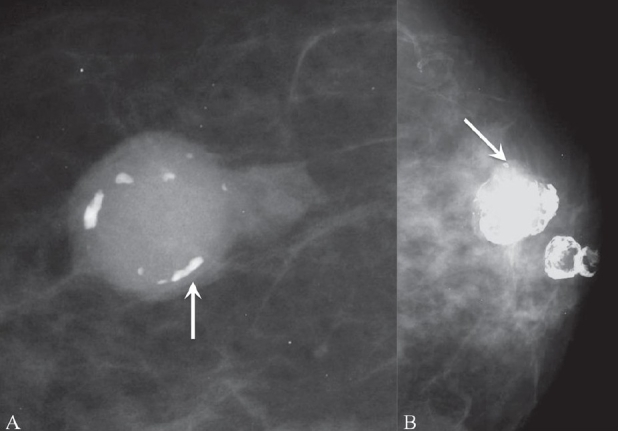
Mammograms show degenerating fibroadenomas with coarse (arrow in A) and popcorn (arrow in B) calcification

These are calcifications seen within degenerating fibroadenomas [Figure 2A and B].

##### Vascular calcifications:

**Figure 3:**
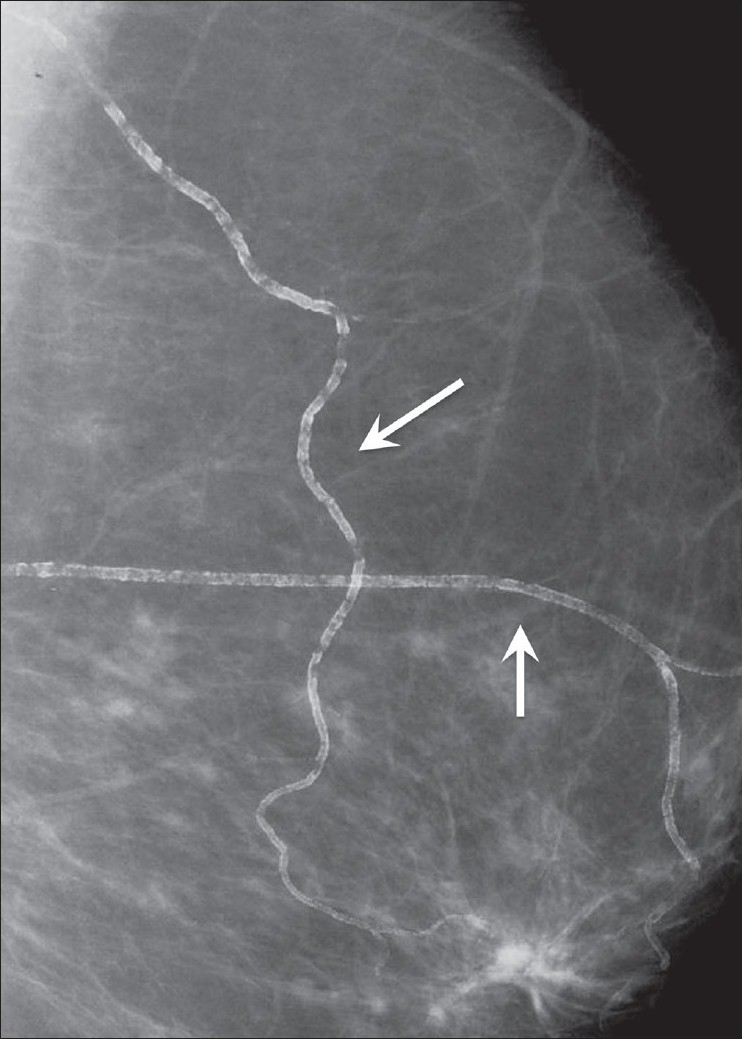
Mammogram shows linear, railroad track calcification (arrows), consistent with vascular calcification

These are also described as railroad track calcifications, showing a linear configuration, either singly or in parallel pairs [Figure 3]. When small, single and linear, these calcifications should be differentiated from malignant calcifications.

##### Large, rod-like calcifications or secretory deposits:

**Figure 4:**
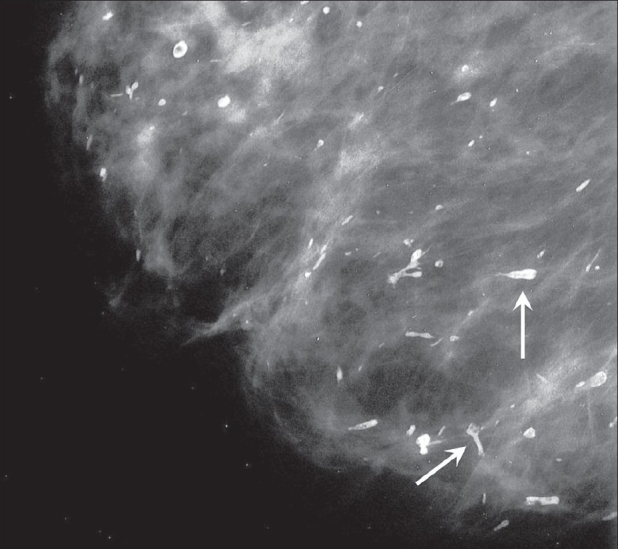
Mammogram shows thick, large, rod-like calcific foci (arrows) due to secretory disease

These are due to secretory disease. The calcific foci are thick and follow the ducts, toward the nipple [Figure 4].

##### Milk of calcium:

**Figure 5:**
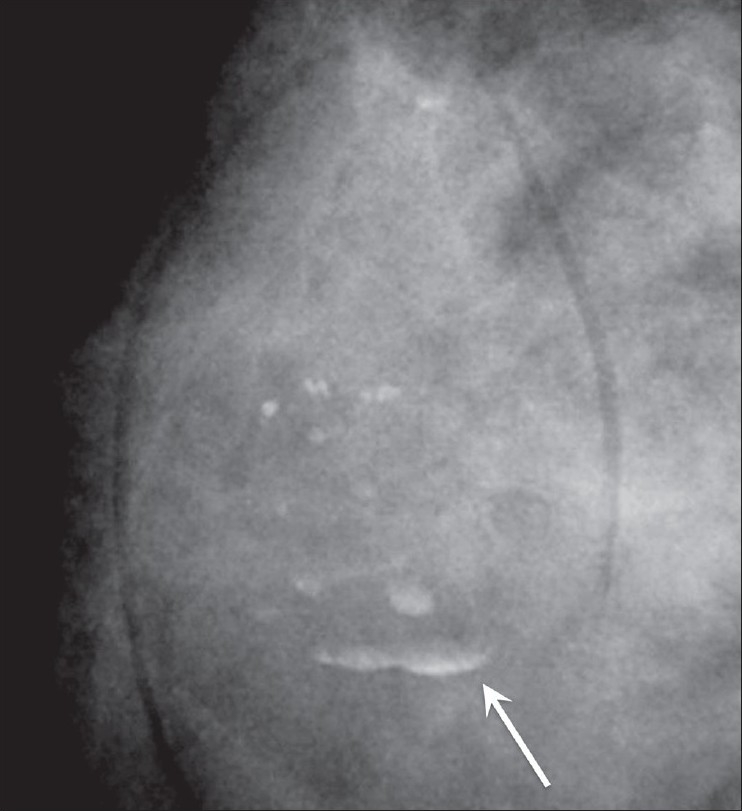
Lateral mammogram shows milk of calcium with layering (arrow)

These are seen as tiny, teacup-shaped calcifications, situated within small cysts on the lateral view [Figure 5]. Sometimes, the small, rounded soft-tissue shadow of the cyst itself is also appreciated.

##### Lucent-centered calcifications:

**Figure 6 (A,B):**
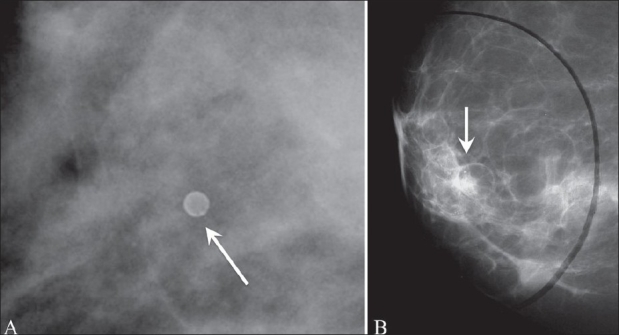
Mammogram (A) shows a lucent-centered focus (arrow) of dermal calcification. Mammogram (B) shows a larger, lucent-centered oil cyst (arrow)

These are rounded calcifications with a lucent center usually representing dermal calcifications [Figure 6A]. Larger calcifications with lucent centers may be due to oil cysts/fat necrosis and may follow surgery or trauma [Figure 6B].

#### Calcifications that are of intermediate concern

##### Amorphous calcifications:

**Figure 7:**
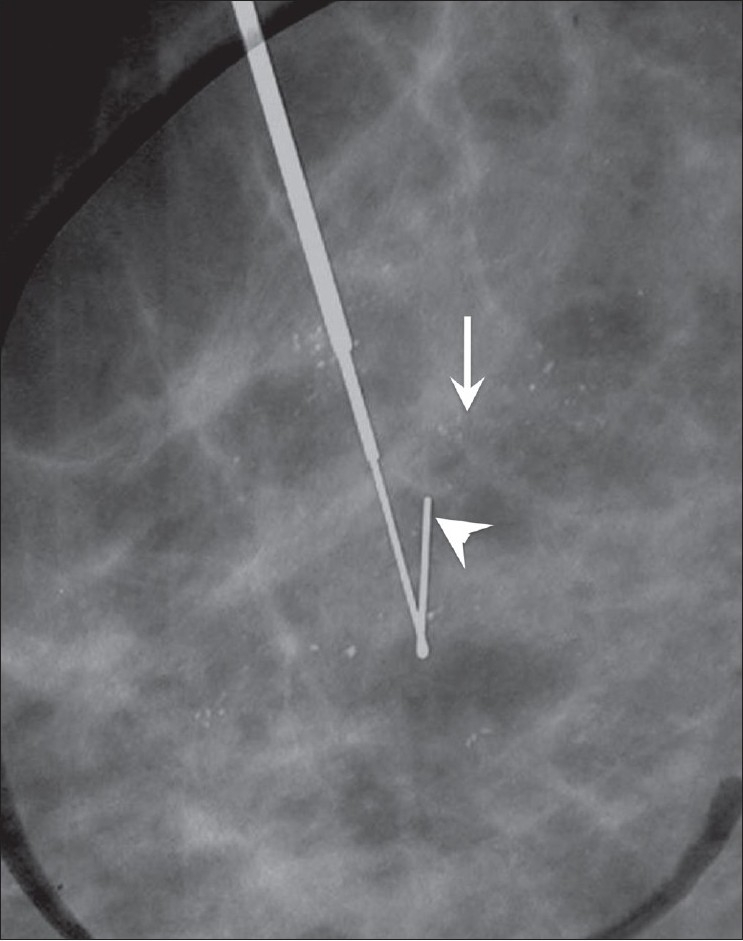
Mammogram shows amorphous calcifications (arrow). A hook-wire localization (arrowhead) was performed; the histopathology report did not show any malignancy

These are very tiny, hazy calcifications [Figure 7] and are often difficult to pick up on CR machines.

#### Calcifications that are highly suspicious for malignancy

##### Fine, linear, branching or casting calcifications:

**Figure 8:**
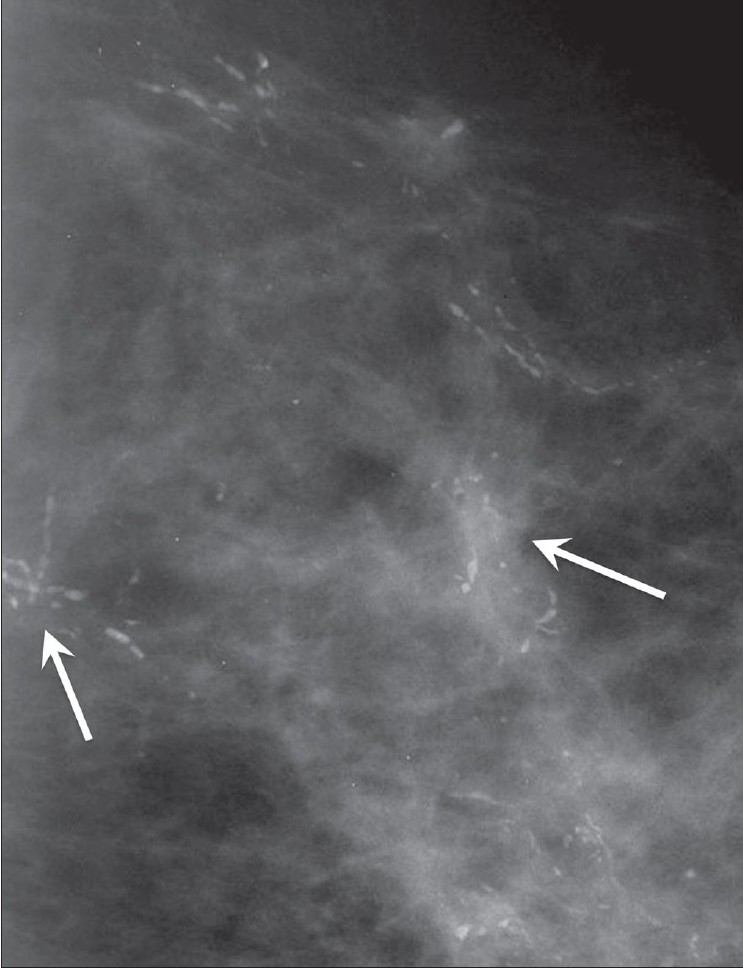
Mammogram shows fine, linear, branching calcifications (arrows), typical of malignancy

These are linear, rod-like calcifications and are typically seen in malignancy [Figure 8].

##### Pleomorphic calcifications:

**Figure 9:**
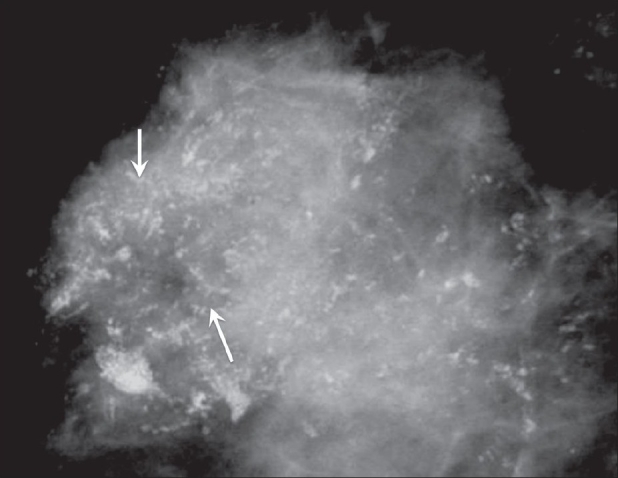
Mammogram shows pleomorphic calcifications (arrows) in this patient with a ductal carcinoma

These are microcalcifications of varying shapes and sizes [Figure 9].

### According to distribution

#### Grouped or clustered:

**Figure 10:**
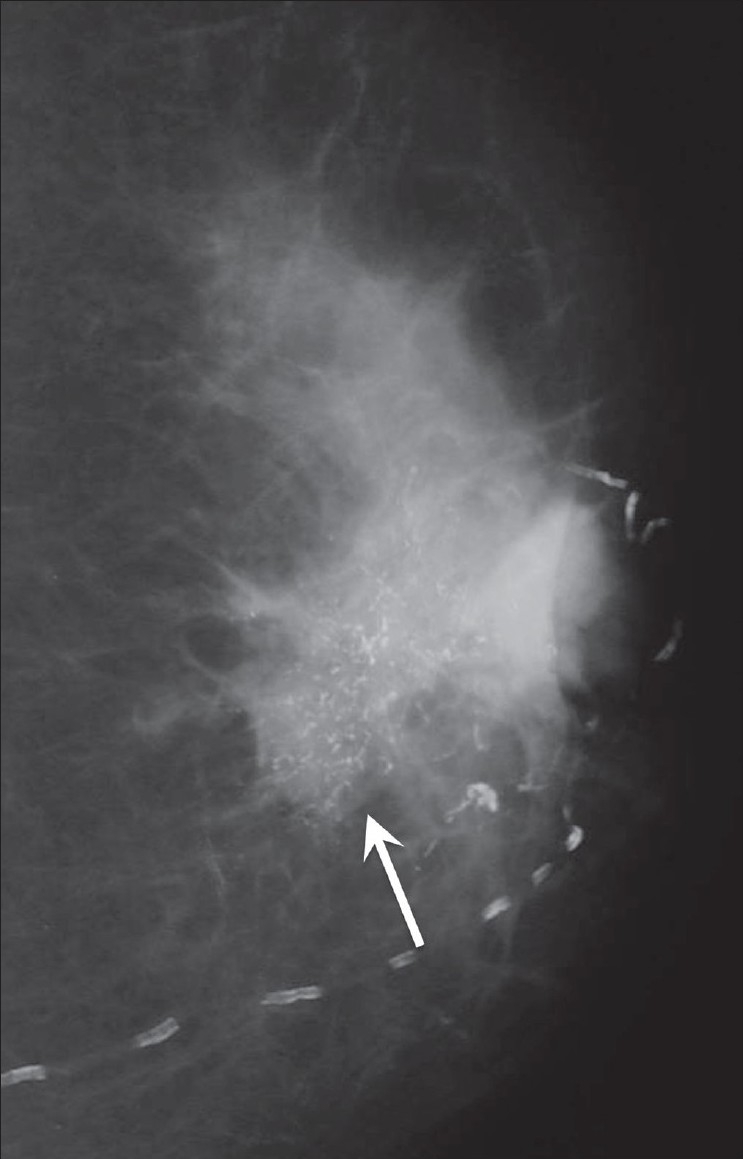
Mammogram shows clustered microcalcifications (arrows)

These are five or more than five calcifications seen in a small area of 1 cm^3^ [Figure 10] and may be seen in benign or malignant conditions. If the cluster is a loose cluster (< 10/cm^2^), it is more likely to represent a benign condition, whereas a compact cluster (>20/cm^2^) is more likely to be due to malignant disease.[6]

#### Linear, segmental:

**Figure 11 (A,B):**
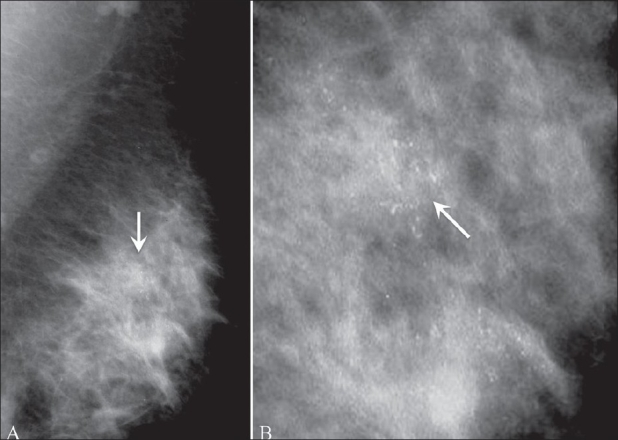
Mediolateral oblique mammogram (A) and magnified view (B) show segmental calcifications

These are suspicious calcifications arranged in a line or showing a branching pattern, suggesting deposits in a duct [Figure 11]. They tend to be distributed in a linear manner because most common malignancies are ductal, beginning in the terminal ducts.

#### Regional:

Calcifications are seen in a large volume, not necessarily conforming to a duct; more likely to be benign.

#### Diffuse or scattered:

**Figure 12:**
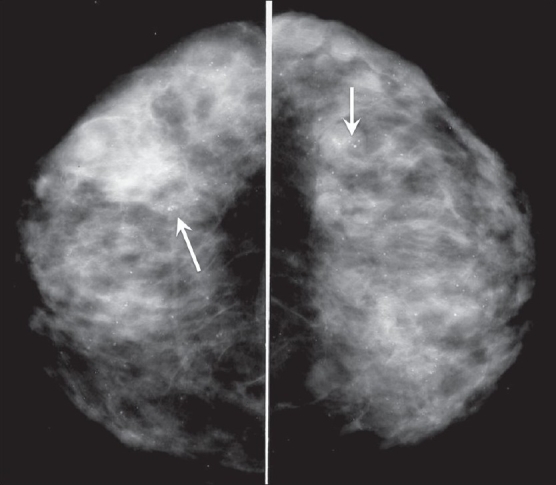
Craniocaudal mammograms of both breasts show benign, diffusely scattered microcalcifications (arrows)

These calcifications are seen all over the breast and may be bilateral [Figure 12]. They are almost always benign.

In conclusion, with the help of morphology and distribution, calcifications can be categorized into benign, of intermediate-concern, and malignant types. It would be more appropriate to categorize them with the help of BI-RADS into 2, 3, 4 and 5.[7] The egg shell, popcorn, lucent-centered, dermal, vascular calcifications, milk of calcium and scattered calcifications are definitely benign and can be categorized as BI-RADS 2. They do not need biopsy or follow-up.

Those of intermediate concern can be categorized into 3 and should be closely monitored. Pleomorphic and casting-type calcifications are categorized as BI-RADS 4 or 5 and a biopsy is recommended. In case follow-up is advised, it should be kept in mind that some microcalcifications, sometimes even of DCIS, can remain unchanged for years. Some calcifications are even known to resolve.[8]
